# Supplementary material for: Systematic prioritization of functional variants and effector genes underlying colorectal cancer risk
Source: Nat Genet. 2024 Sep 16;56(10):2104–11. doi: 10.1038/s41588-024-01900-w (PMC11525171; doi:10.1038/s41588-024-01900-w)
Supplement: Supplementary file 1 — Supplementary Methods and Supplementary Figs. 1–10 [file 41588_2024_1900_MOESM1_ESM.pdf]

nature genetics

Supplementary information https://doi.org/10.1038/s41588-024-01900-w

Systematic prioritization of functional variants and effector genes underlying colorectal cancer risk

In the format provided by the authors and unedited

SUPPLEMENTARY METHODS

ChIPmentation

ChIPmentation was performed on histone marks H3K4me1 (C15410194, Diagenode) H3K4me3 (C15410003, Diagenode), H3K27ac (C15410210, Diagenode), H3K27me3 (C15410195, Diagenode), H3K36me3 (C15410192, Diagenode) and CTCF (C15410210, Diagenode) for C32, CL11, HT29, SW403, SW480, SW948 cell lines using the published protocol[1], with minor modifications.

3-4 x 10^7 cells were fixed for 10 mins in 1% methanol-free formaldehyde and quenched with 200mM glycine. Samples were washed in PBS-A and the pellets flash frozen in LN_2 to be stored in -80°C until use. During the experiment, cell pellets were thawed and lysed on ice. From the pellets, 10^7 cells were resuspended in 10ml of ice-cold cytoplasmic lysis buffer (50mM HEPES, 150mM NaCl, 1mM EDTA, 1% Triton X-100, 0.1% sodium deoxycholate [NaDOC], 0.1% SDS) with protease inhibitors, incubated in a cold room for 15 mins, and pelleted at 1,200rpm, 4°C. This step was repeated twice. The pellet was incubated in a high SDS lysis buffer (50mM HEPES, 150mM NaCl, 1mM EDTA, 1% Triton X-100, 0.1% NaDOC, 1% SDS) for 15 min and re-pelleted. The pellet was resuspended in cold sonication buffer (10mM Tris, 1mM EDTA, 0.1% SDS) and the chromatin was sonicated for 15 mins using a Covaris E220 in a 1ml Covaris Millitube containing 2-3 x 10^7 cells. The lysates were diluted using a ChIP dilution buffer (50mM Tris, 225mM NaCl, 0.15% NaDOC, 1.5% Triton X-100) in a ratio of 1:2 to neutralise SDS. Chromatin lysates were pre-cleared using Protein G Dynabeads (ThermoFisher Scientific) prior to the addition of 5μg of target antibody (Diagenode) per 3 x 10^5 cells. 10% of input was frozen for input controls. After overnight incubation at 4°C with antibody, 30μl Protein G Dynabeads were added and incubated for 2 hrs, and sequentially washed in low salt, high salt, LiCl, and Tris buffer three times each. Each sample was tagmented using loaded Tagmentase bound with sequencing adaptors (Diagenode) whilst still bead bound for 10 mins at 37°C and 1,400 rpm. The washed beads were resuspended in ChIP elution buffer and reverse cross-linked overnight using Proteinase-K at 65°C. DNA was extracted using a phenol-chloroform extraction method and barcoded NGS libraries for each sample were prepared using UDI for Tagmented Libraries (Set I-II; Diagenode) and Kapa HiFi PCR master mix (Roche). Libraries were cleaned and size selected using Kapa Pure beads (Roche). The libraries were analysed using an Agilent Bioanalyzer DNA high sensitivity chip (Agilent Technologies). The final pooled library was quantified using a NEB library quant kit (NEB) on a QuantStudio7 Real Time PCR (Thermo Fisher). The input controls were treated in the same manner, but without the addition of antibody. ChIPmentation libraries were generated using the end repair and adaptor protocol with the NEBnext Library Prep Kit (NEB) and sequenced as 100bp single end reads to a desired depth of 50-100 million reads per library using a NovaSeq 6000 (Illumina). Data processing was performed using the Nextflow nf-core chipseq pipeline v1.2.1[2] using default parameters.

Omni-ATAC

ATAC-seq was performed on the C32, CACO2, CL11, HT29, SW403, SW480, SW948 MSS CRC cell lines and HCEC-1CT normal colon cell line, as per Corces *et al* (2017)[3]. 5 x 10^5 cells with >90% viability were pelleted and resuspended in ice cold RSB buffer containing 0.1% NP40, 0.1% Tween 20 and 0.1% Digitonin, and incubated for 3 mins. The lysis was stopped using 1ml of cold RSB buffer. Nuclei were pelleted and resuspended in a transposition mix in a 100nM final concentration of loaded transposase (Diagenode). The samples were tagmented at 37°C for 30 mins on a thermomixer

at 1,000 rpm shaking. Eluted DNA were amplified using UDI for Tagmented Libraries (Set I-II; Diagenode) and Kapa HiFi PCR master mix (Roche) for 7-10 cycles. The libraries were analysed using an Agilent Bioanalyzer DNA High Sensitivity Chip. The final pooled library was quantified using a Library Quant Kit (NEB) on a QuantStudio7 Real Time PCR (Thermo Fisher). Each library was sequenced as 50bp pair end reads, performed using a NovaSeq 6000 (Illumina) to a depth of 70-100 million reads. Data processing was performed using the Nextflow nf-core atacseq pipeline v1.2.1[4] using default parameters. Peaks for both the ChIP-seq and ATAC-seq were consistent across all cell lines (**Supplementary Fig. 13**).

Micro-C

We generated Micro-C chromatin interaction maps of CL11, HT29, SW403, SW480, and SW948 cell lines as *per* Krietenstein *et al* (2020)[5] and Hsieh *et al* (2020)[6]. Cells were fixed at a density of 10^6 cells/ml in 3mM DSG for 20 mins at room temperature. After addition of methanol-free formaldehyde to a final concentration of 1% for 10 mins, reactions were quenched by addition of glycine to a final concentration of 600nM for 5 mins, washed in 1x PBS then snap frozen in LN_2. Fixed cells were digested with micrococcal nuclease (MNase, Worthington) optimised for each cell line and batch, with incubation for 10 mins at 37°C, 1,000 rpm. Reactions were quenched by EGTA at a final concentration of 17.5nM, with incubation for 10 mins at 65°C, 1,000 rpm. Samples were pooled such that each replicate library had 2.5-3 x 10^6 cells to ensure adequate library complexity. End repair and biotin labelling was performed by incubation of 10^6 MNase-digested cells using 30U of T4 PNK (NEB) at 37°C for 15 mins, 1,000 rpm. 35U Large Klenow Fragment (NEB) was added and incubation at 37°C for 15 mins, 1,000 rpm performed. After addition of biotinylated dATP (Biotin-14-dATP, NU-835-BIO14-S; Jena Biosciences), biotinylated dCTP (Biotin-14-dCTP, NU-956-BIO-14-S; Jena Biosciences), dTTP and dGTP (each 66µM final concentration), samples were incubated at 25°C for 45 mins, 1,000 rpm. Reactions were stopped using 40mM EDTA and heating to 65°C for 20 mins. Ligation was carried out with 10,000U T4 DNA ligase, 23°C for 3 hrs, 1,000 rpm. Biotin ends were excised using 200U Exonuclease III at 37°C for 10 mins, 1,000 rpm. Samples were incubated overnight at 65°C with Proteinase K to reverse crosslink the DNA, followed by elution using phenol-chloroform and run on a 2.5% TAE gel. Bands within 250-400bp were excised and purified, and biotinylated DNA capture performed using C1 streptavidin beads (Invitrogen). The DNA bound beads were end repaired and adaptor ligated using NEBnext Library Prep Kits (NEB). Finally, eluted DNA was amplified using NEB Unique Dual Index primers to produce final libraries. Size selection was performed using Kapa Pure beads (Roche). Libraries were quantified using Agilent Bioanalyzer DNA High Sensitivity Chips and NEB Library Quant Kits. As a quality check before deep sequencing, the pooled library consisting of eight individual replicates was run on a MiSeq v2. The data was analysed using JuicerTools[7] to count valid interactions. We required >90% valid interactions to be *cis*-contacts, of which 60-70% had to be short range *cis*-contacts. If the metrics were satisfactory, the pooled library was sequenced on a NovaSeq 6000 (Illumina) to a depth of at least 300 million reads per library, 100bp paired end sequencing.

We used the nf-distiller pipeline[8] v0.3.4 to generate the interaction maps from raw FASTQ files, using matrix balancing normalisation and binned at 1kb. FitHiC2[9] was used to call significant interactions, merging adjoining bins with significant interactions. TADs and compartments were identified using cooltools[10] v0.5.4, utilising 30kb and 100kb windows, respectively, and binning at 10kb. Compartments were determined using an eigendecomposition of the

contact matrix. The GC content of each bin was used as a phasing track. Active compartments are defined as having the value of the first eigenvector was positive, and inactive if it was negative.

RNA extraction and library sequencing

The RNA sequencing of C32, CL11, HT29, SW403, SW948, and HCEC-1CT was performed. RNA was extracted from 10^6 cells using the RNeasy Mini Kit (Qiagen) and quantified by Qubit 3.0 fluorometer (Life Technologies) with the RNA Quantification Broad Range kit (Invitrogen), and the 2100 Bioanalyser (Agilent), using the RNA 6000 Nano Reagents (Agilent). All the samples had RNA integrity number >9.3. Samples were barcoded for library preparation with ribosomal RNA depletion and sequenced using an Illumina NovaSeq 6000 (100bp paired-end, 100 million reads per library). Analysis of RNA-seq data was performed using the RNAflow pipeline[11] v1.4.1 using default parameters.

SUPPLEMENTARY FIGURES

A

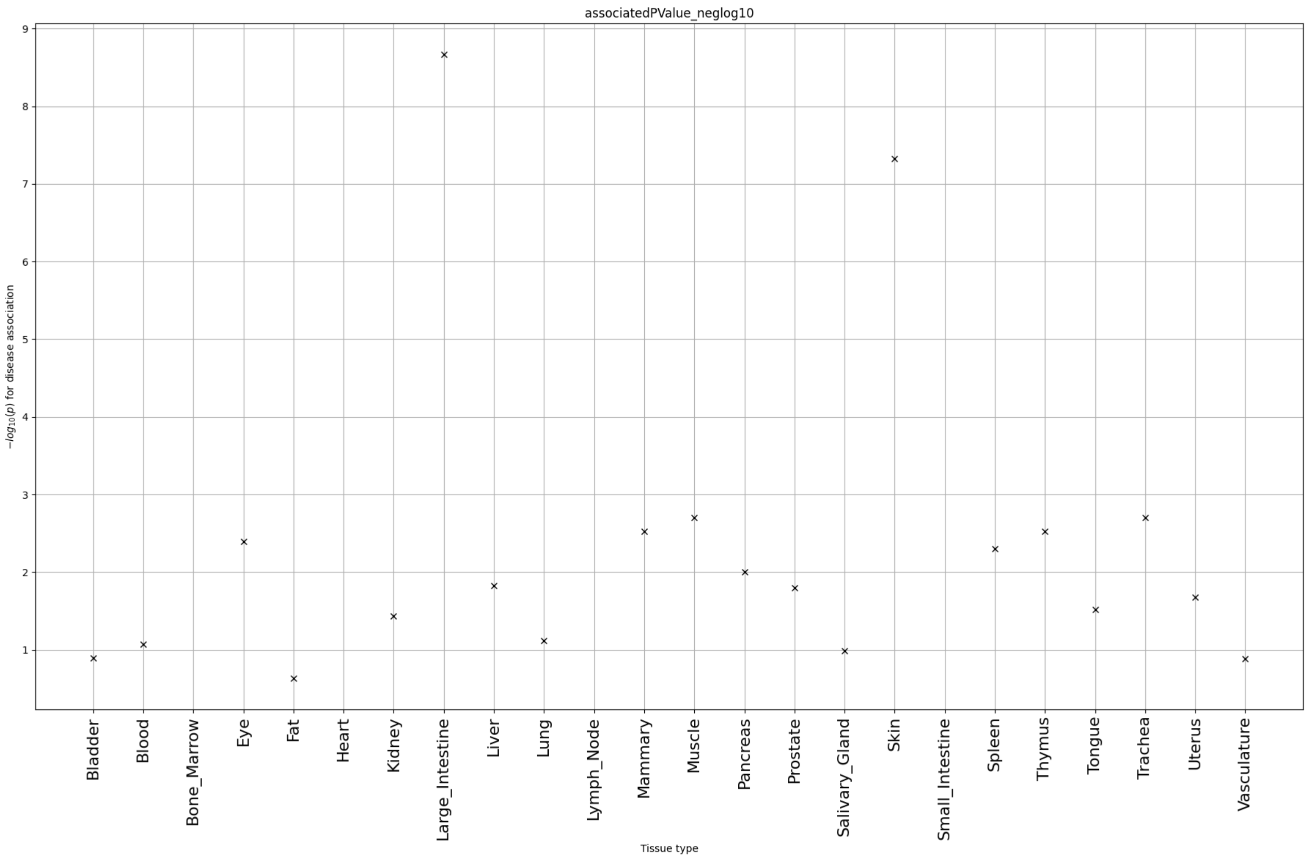

B

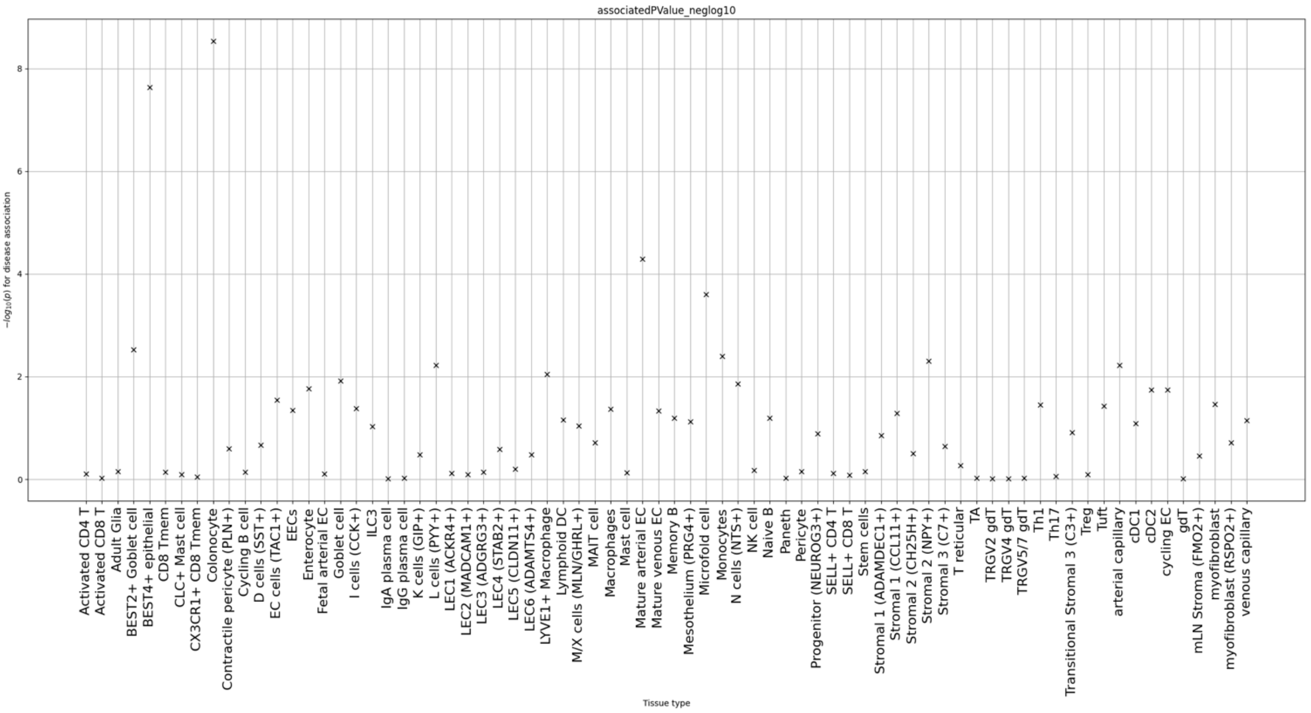

Supplementary Figure 1: Enrichment plots from the scDRS[12] analysis in (A) 24 different tissues from the Tabula Sapiens dataset[13], and (B) different cell types from the colon from the Gut Cell Atlas[14]. Enrichment *P*-values were calculated empirically by comparing the disease scores to the pooled control scores.

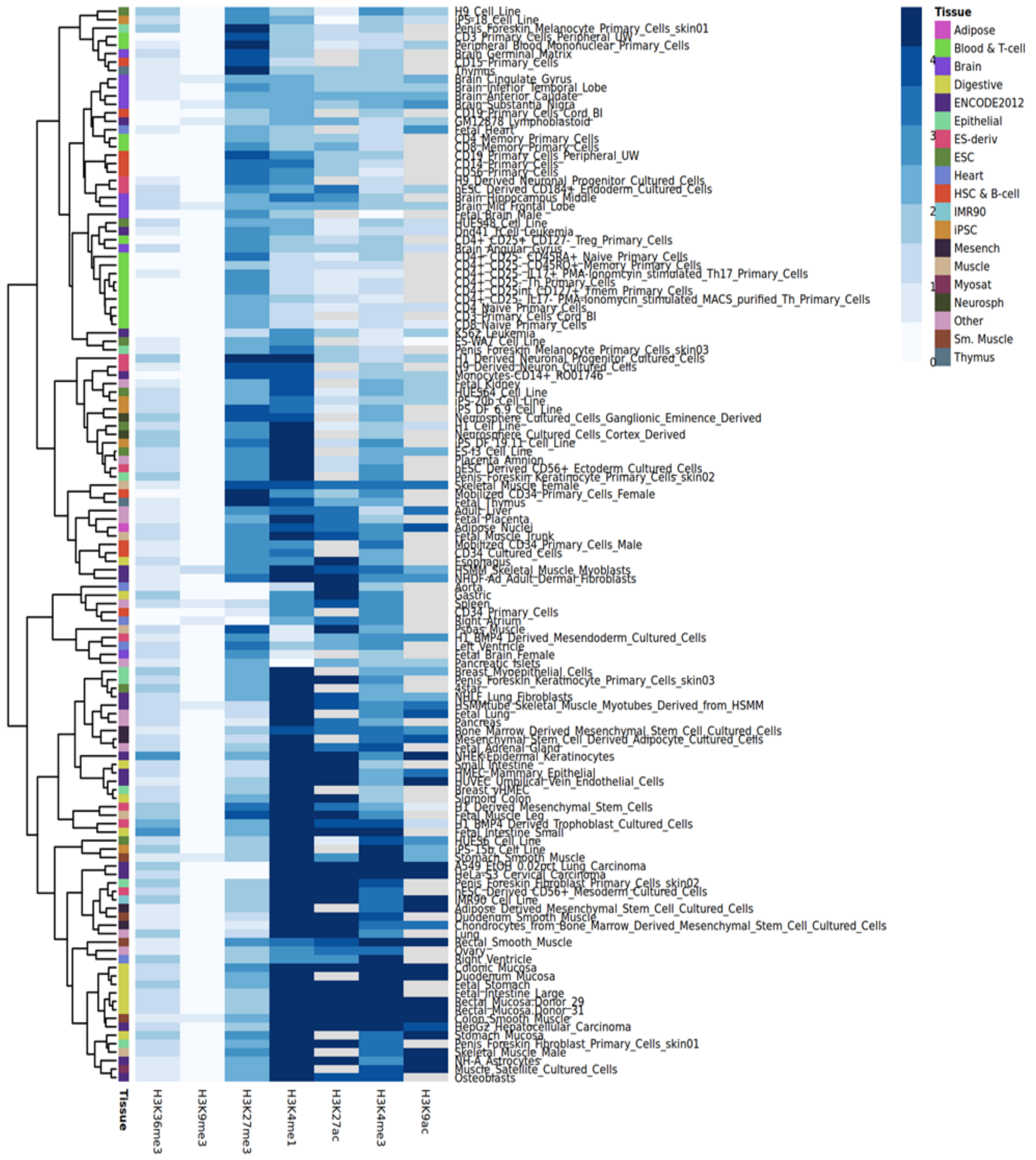

Supplementary Figure 2: Heatmap of enrichment scores for the GWAS variants in a range of histone marks in multiple tissue types from the NIH Roadmap Epigenomics Project[15]. Darker colours indicate a more significant enrichment *P*-value. The colour bar on the left indicates the tissue category. Enrichment *P*-values were calculated empirically using 50,000 permutations of randomly selected variants.

A

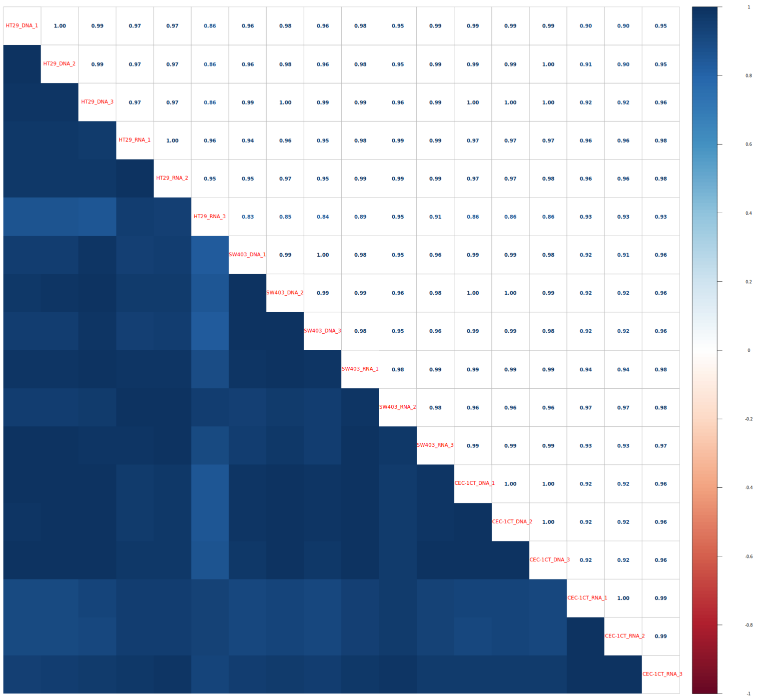

B

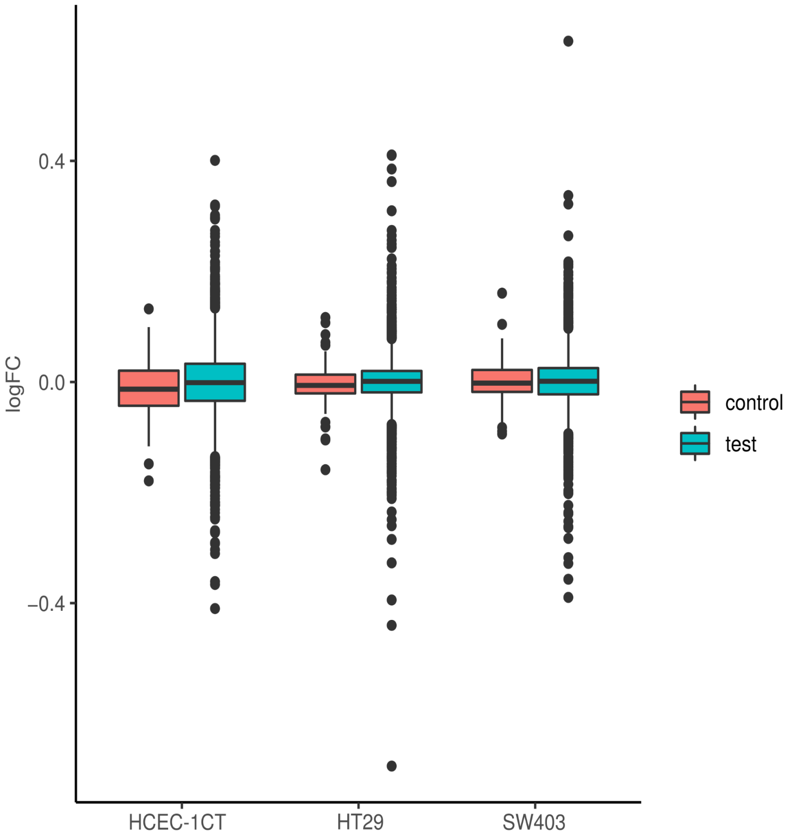

Supplementary Figure 3: Quality control of the MPRA data. (A) Correlation of mapped reads between replicates and cell lines. Pearson correlation coefficients are shown in the upper triangle. (B) log_2fold-change between the control sequences (in red, n=98) and the test sequences (in blue, n=5,281 for all cell lines). The lower and upper edges of the boxes correspond to the first and third quartiles, and the middle line represents the median. The whiskers represent 1.5x the distance between the first and third quartiles from the box. Points outside this range are plotted individually.

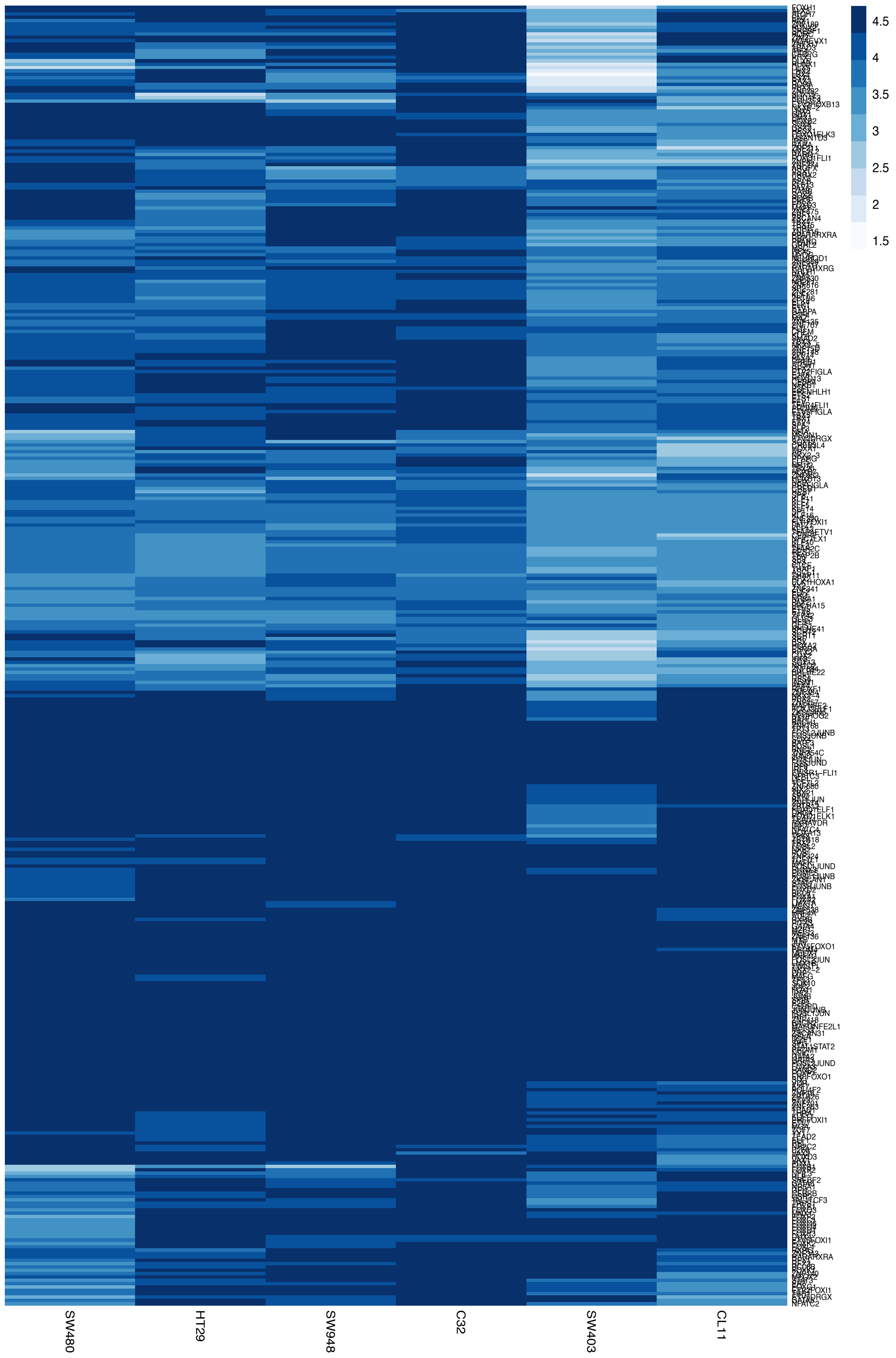

Supplementary Figure 4: Heatmap of the enrichment of transcription factors in the GWAS regions. Darker colours indicate a more significant enrichment *P*-value. Enrichment *P*-values were calculated empirically using 50,000 permutations of randomly selected variants.

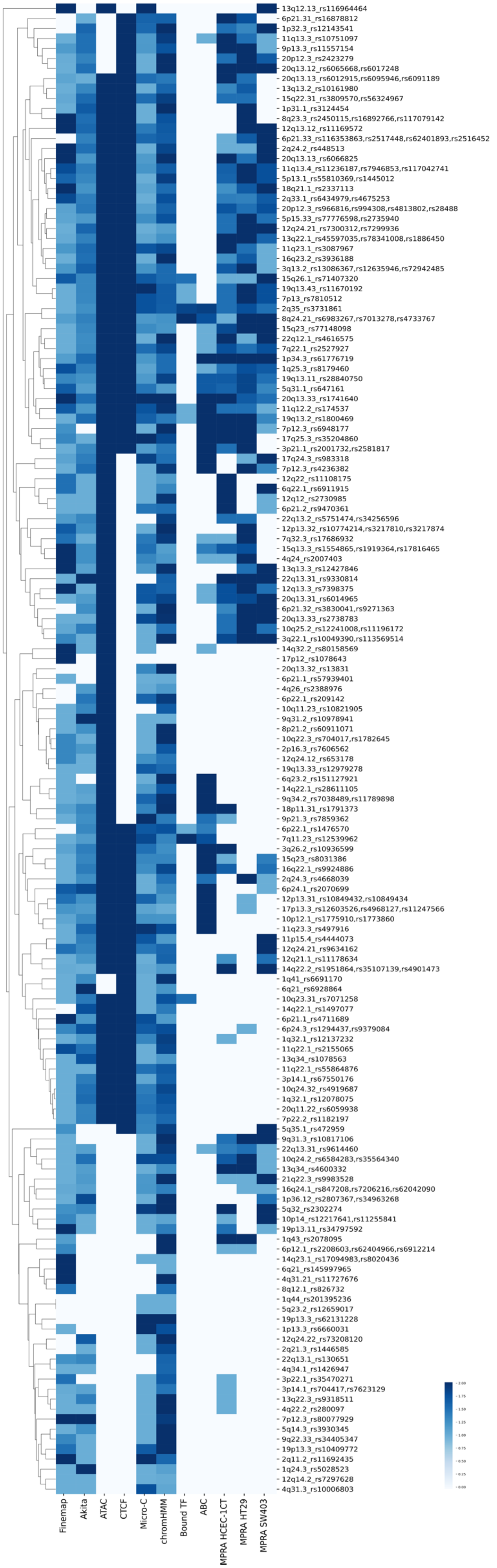

Supplementary Figure 5: Heatmap of the annotations for each GWAS locus. Annotation scores were averaged across the variants in the locus. Darker blue colours indicate stronger evidence of the annotation being present in the locus. GWAS loci that overlapped were merged.

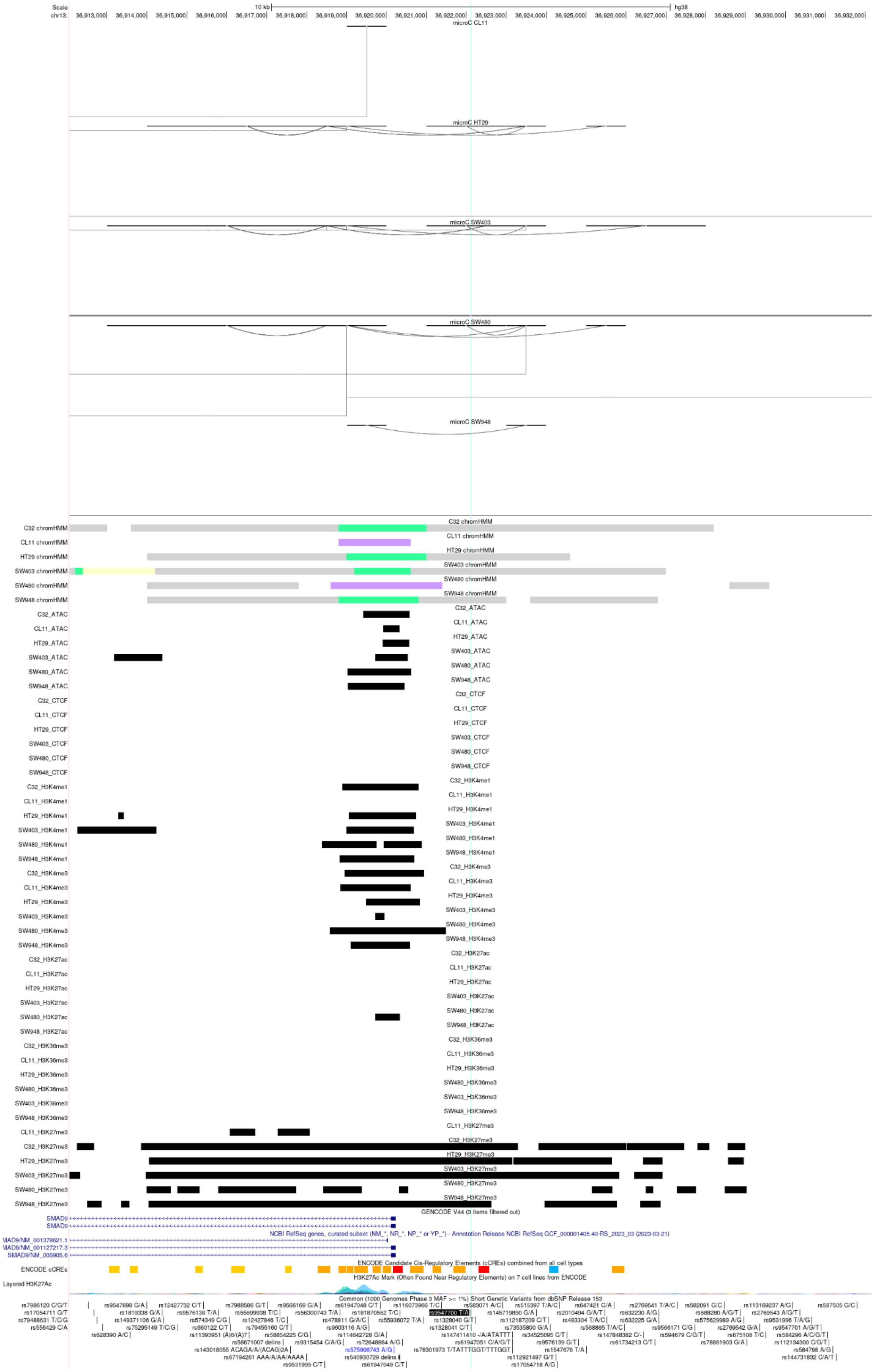

Supplementary Figure 6: Detailed annotation for the variants in 13q13.3 locus from UCSC Genome Browser. The putative variant, rs9547700, is highlighted in light blue. ChromHMM annotations are coloured as per Fig. 3. Annotation data for all the GWAS regions are available on UCSC Genome Browser (https://genome.ucsc.edu/s/philip.law%40icr.ac.uk/CRC%20GWAS%20annotation).

A

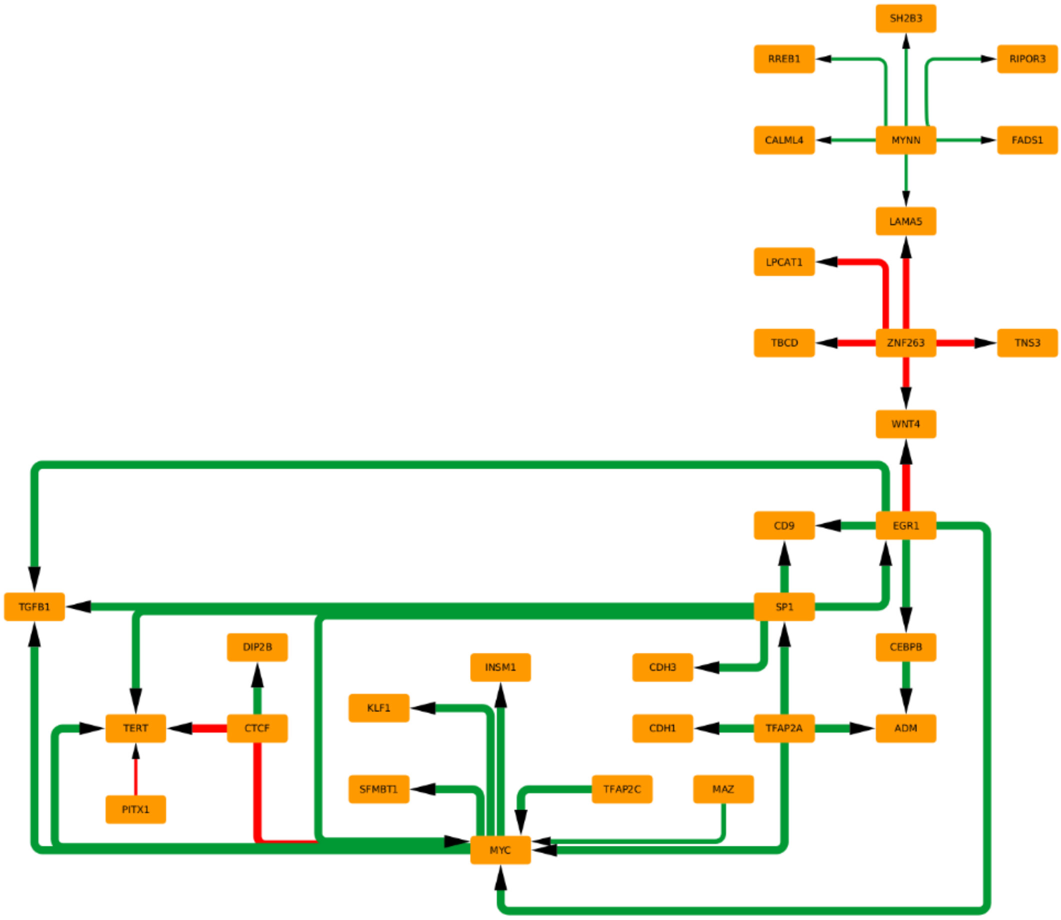
SH2B3
RREB1
RIPOR3
CALML4
MYNN
FADS1
LAMA5
LPCAT1
TBCD
ZNF263
TNS3
WNT4
CD9
EGR1
TGFB1
SP1
DIP2B
INSM1
CDH3
CEBPB
KLF1
TERT
CTCF
CDH1
TFAP2A
ADM
PITX1
SFMBT1
TFAP2C
MAZ
MYC

B

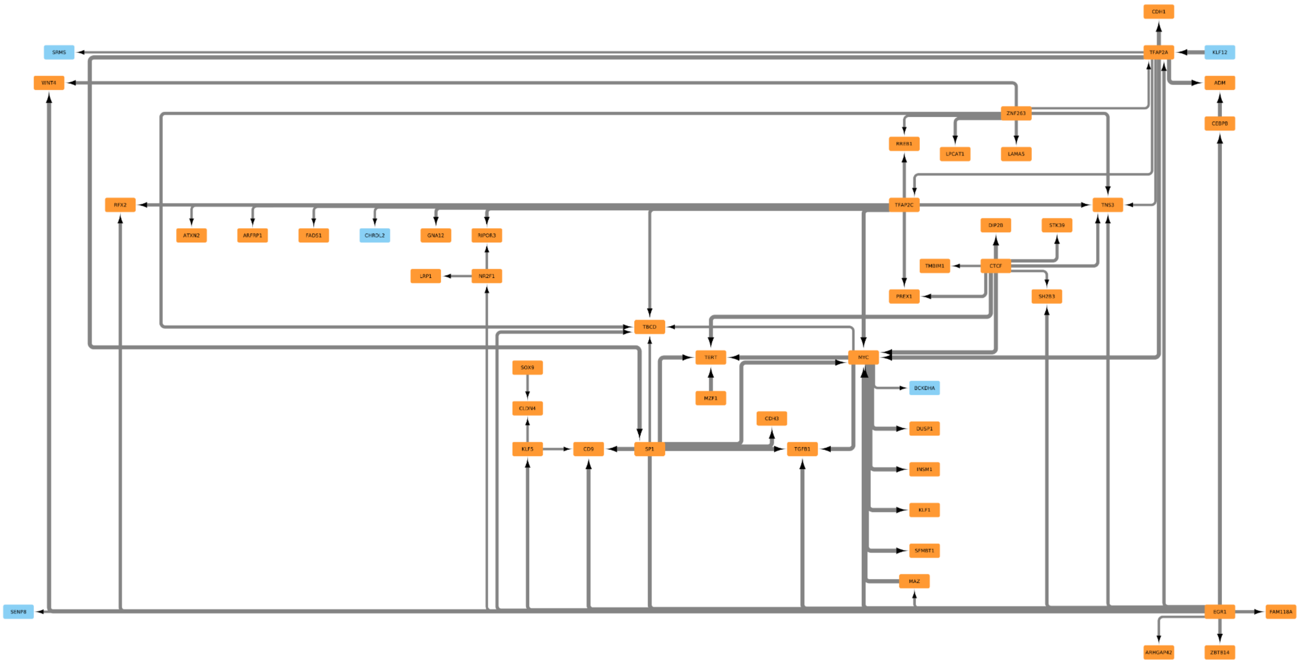

C

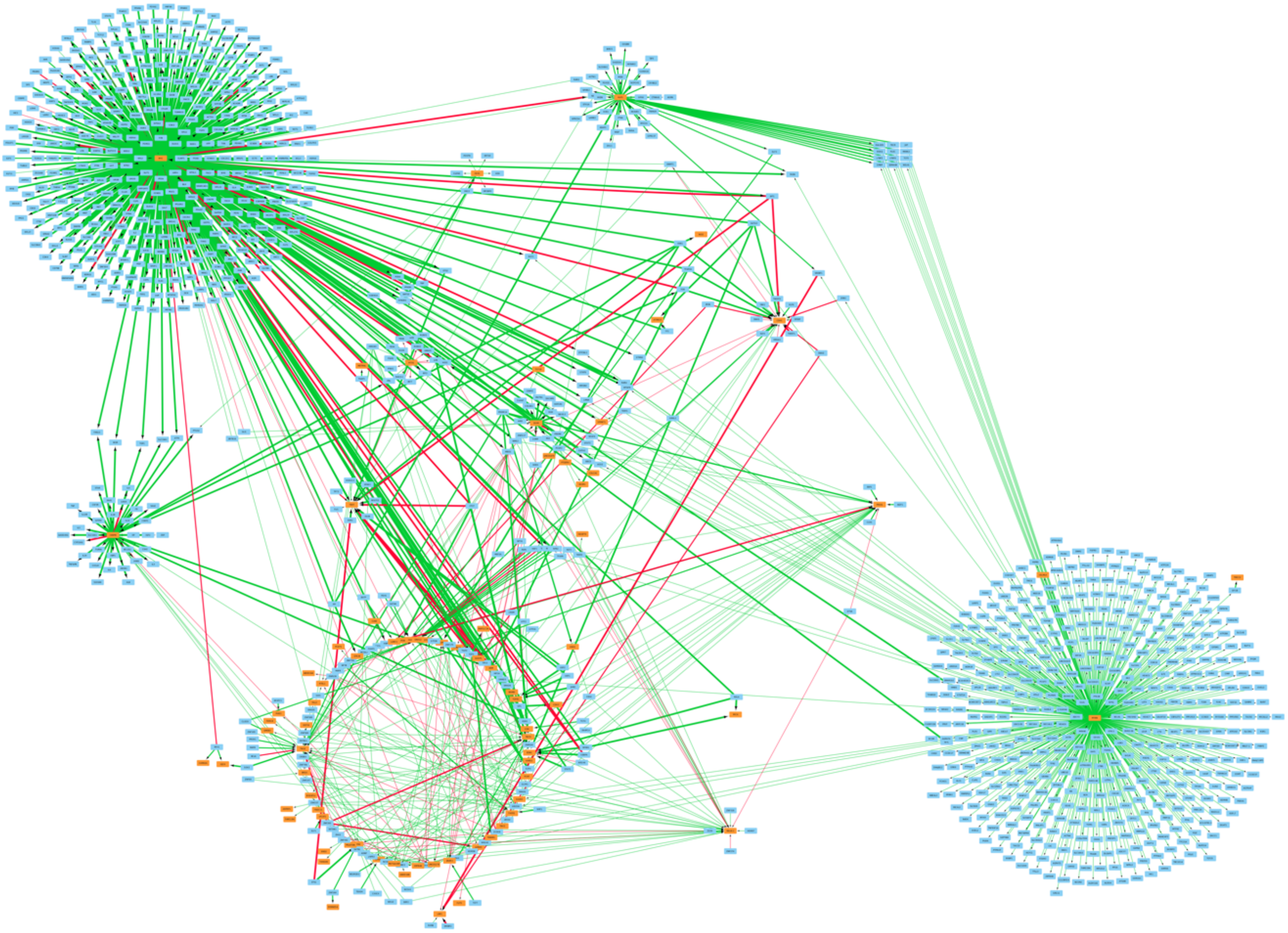

Supplementary Figure 7: Regulatory map of the genes and TFs identified in this analysis. Green lines indicate stimulation and red lines indicate repression. Line thickness is indicative of the confidence of the interaction. Regulatory interaction data were obtained from the OmniPath[16]/DoRothEA[17]. (A) Associations derived from cancer cells. (B) Associations derived from all cells. (C) Associations in cancer tissues, where only one gene interaction were present in the gene list. Genes present in the gene list are coloured orange. In Figure A and B, both genes in the interaction had to be present in the gene list.

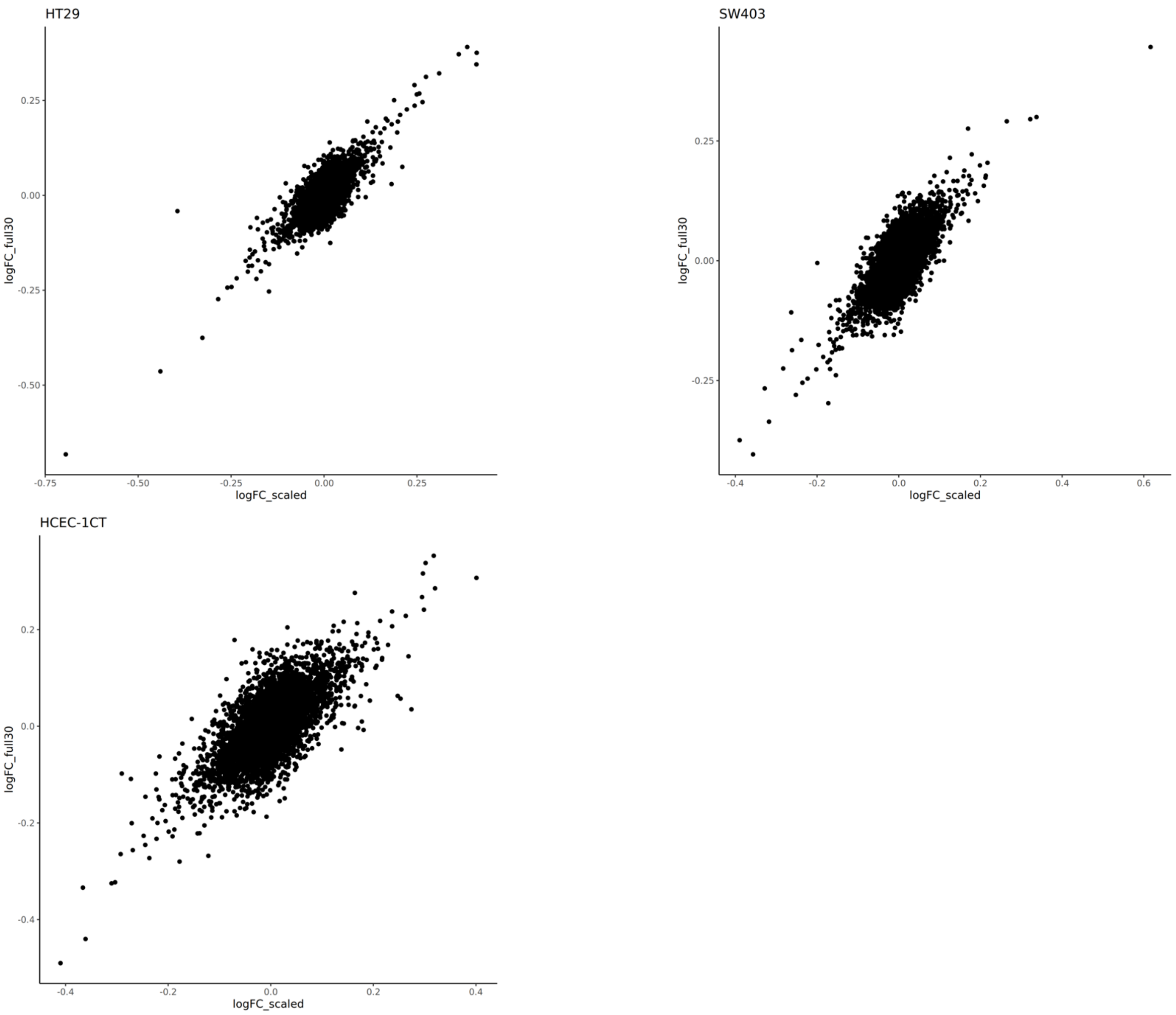

Supplementary Figure 12: Consistency of MPRA log fold changes between using the scaled model and using the full model on down-sampled data. Data were down-sampled to have 30 barcodes per variant. Pearson correlation: 0.76, 0.75, 0.74 for HT29, SW403, and HCEC-1CT, respectively.

ATAC-seq peaks

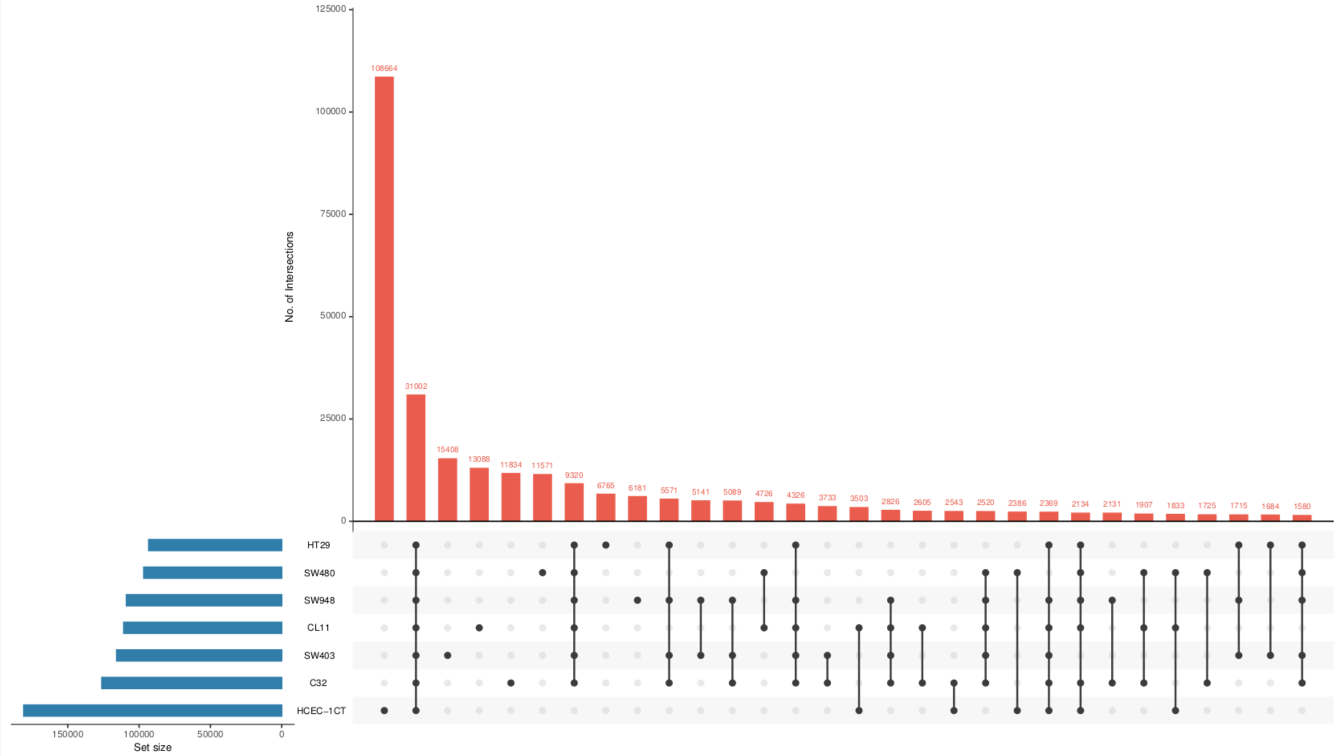

CTCF peaks

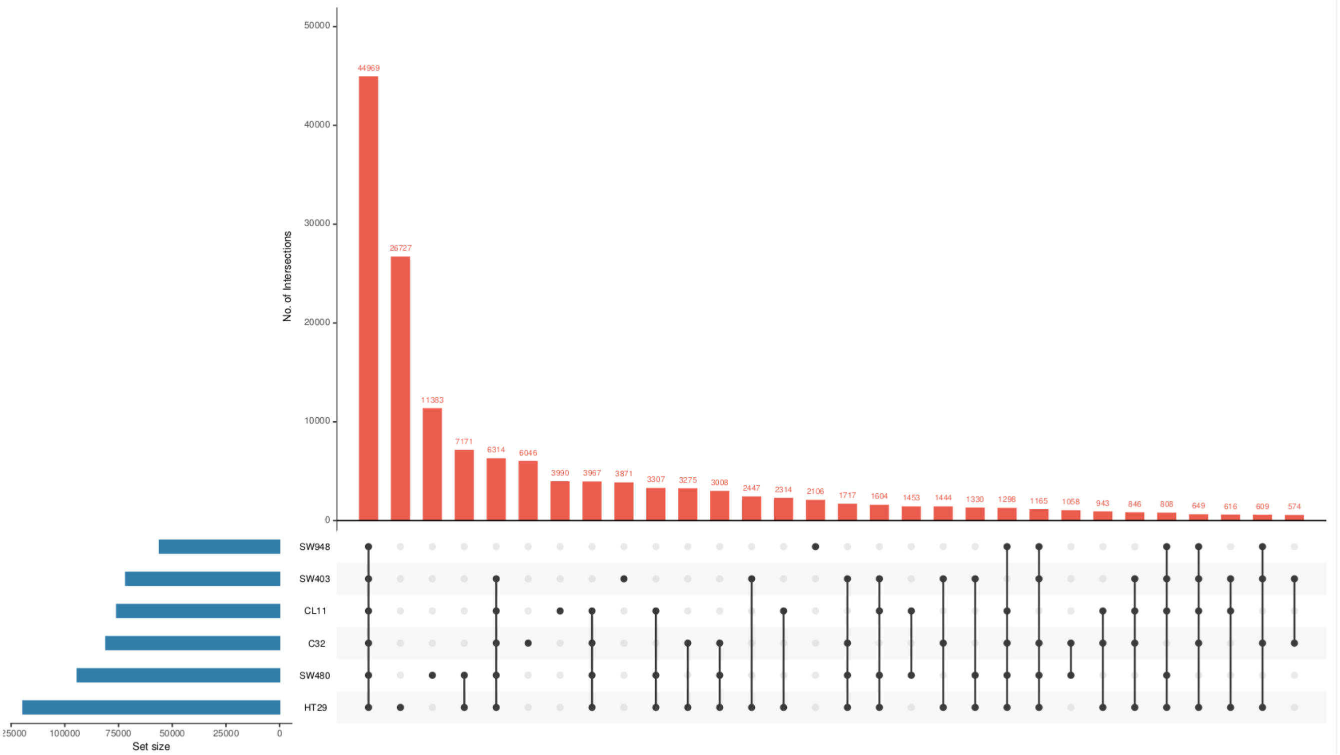

H3K4me1 peaks

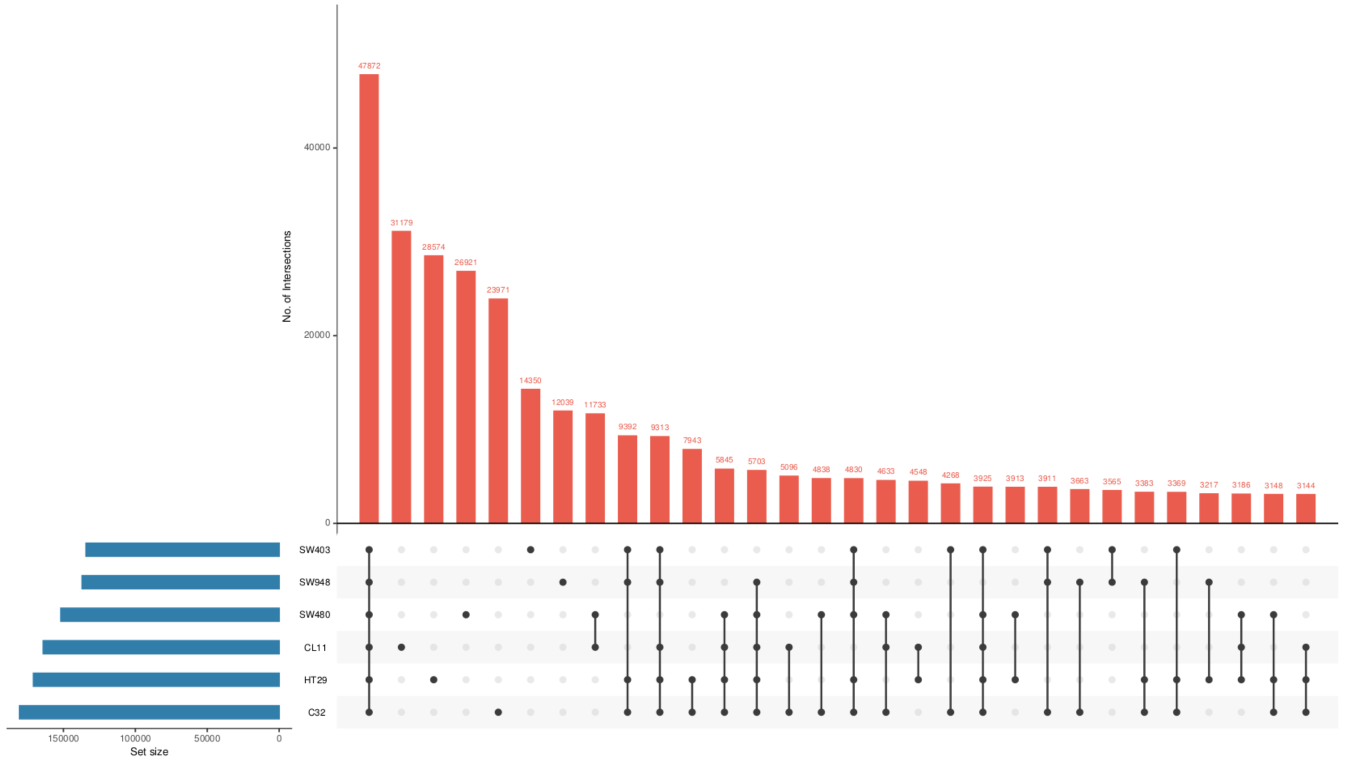

H3K4me3 peaks

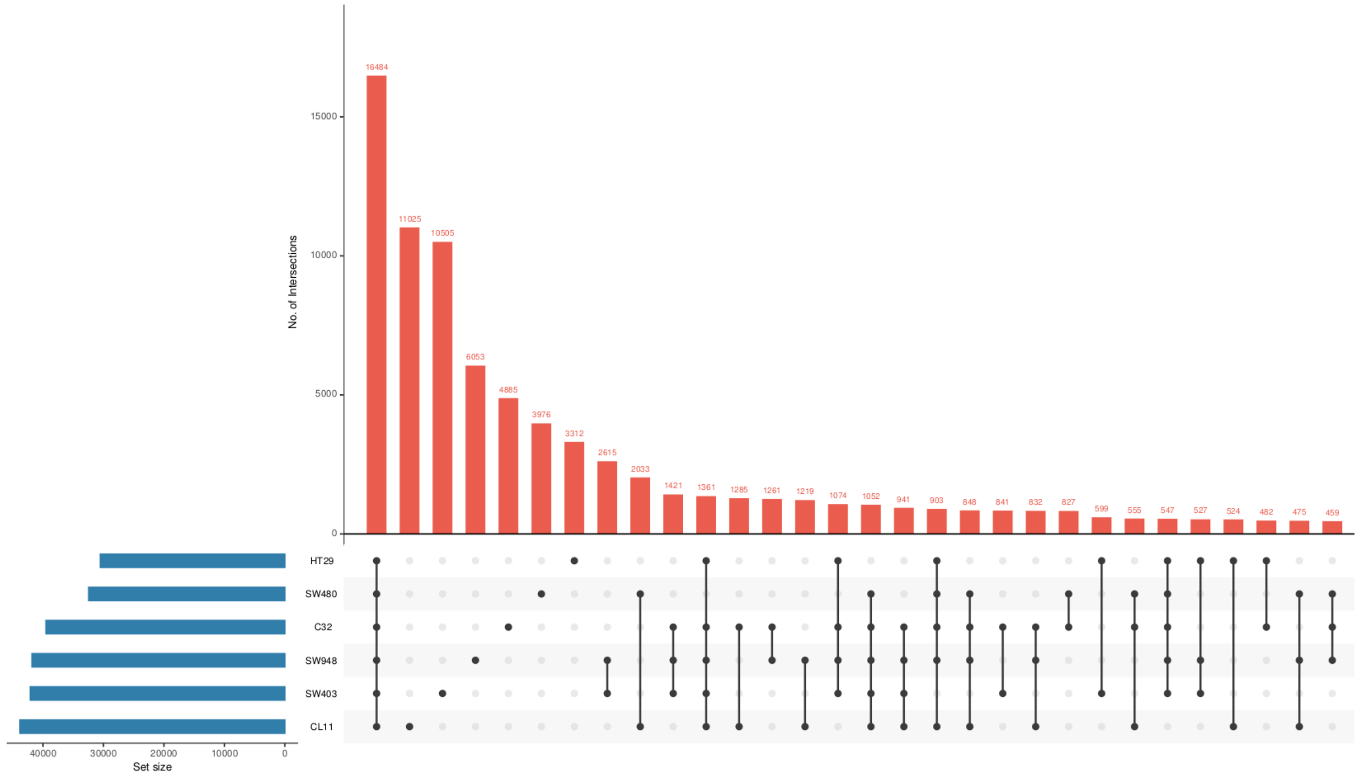

H3K27ac peaks

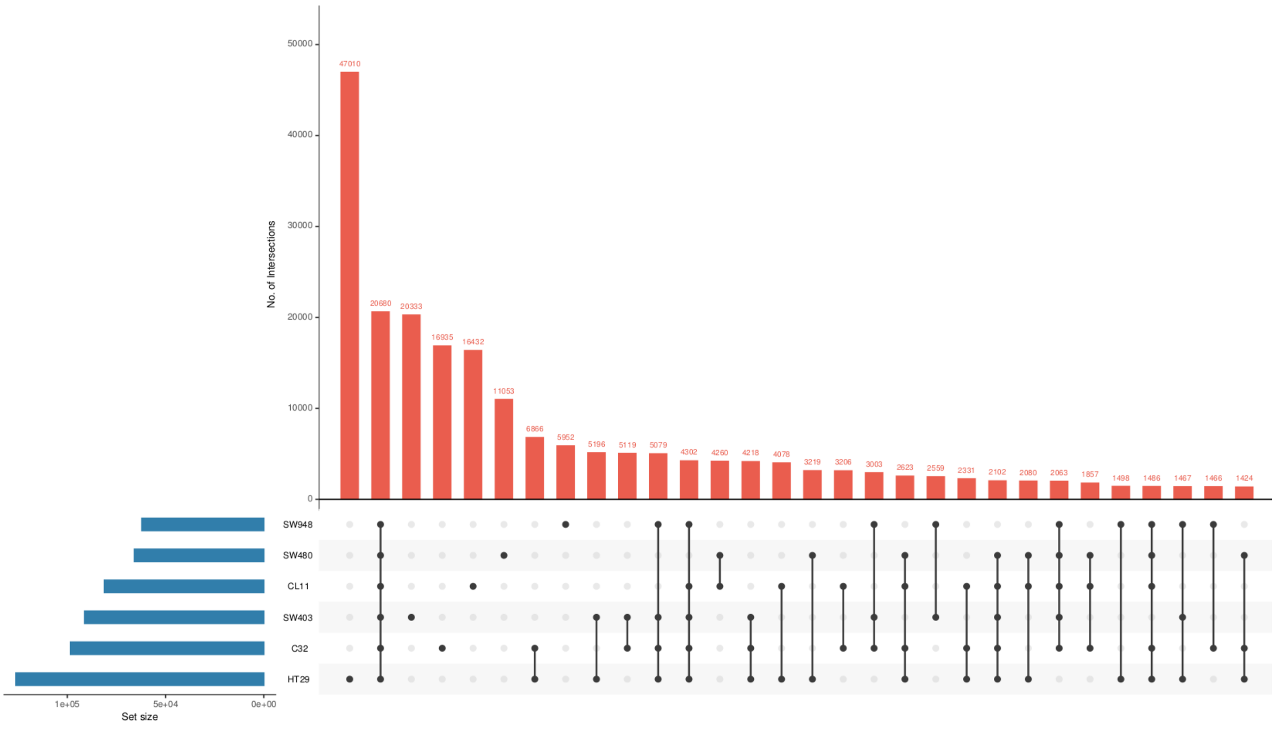

H3K27me3 peaks

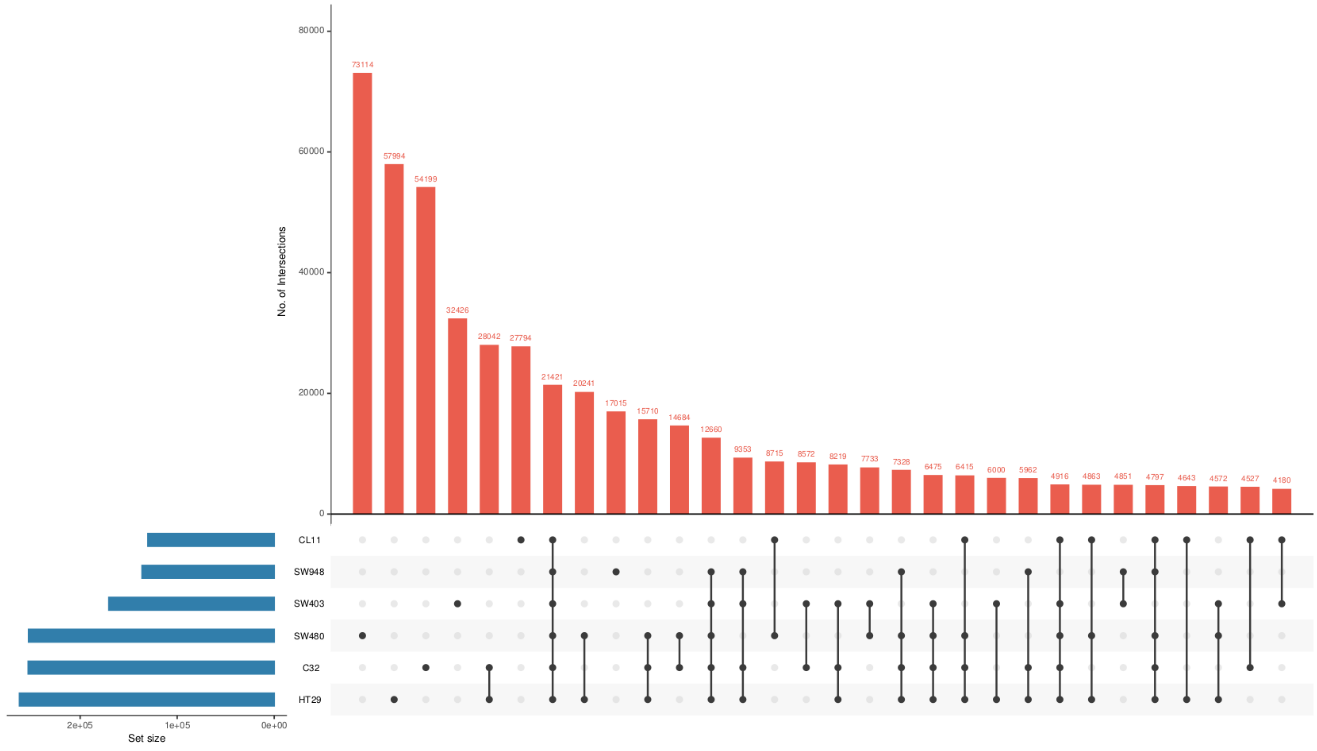

H3K36me3 peaks

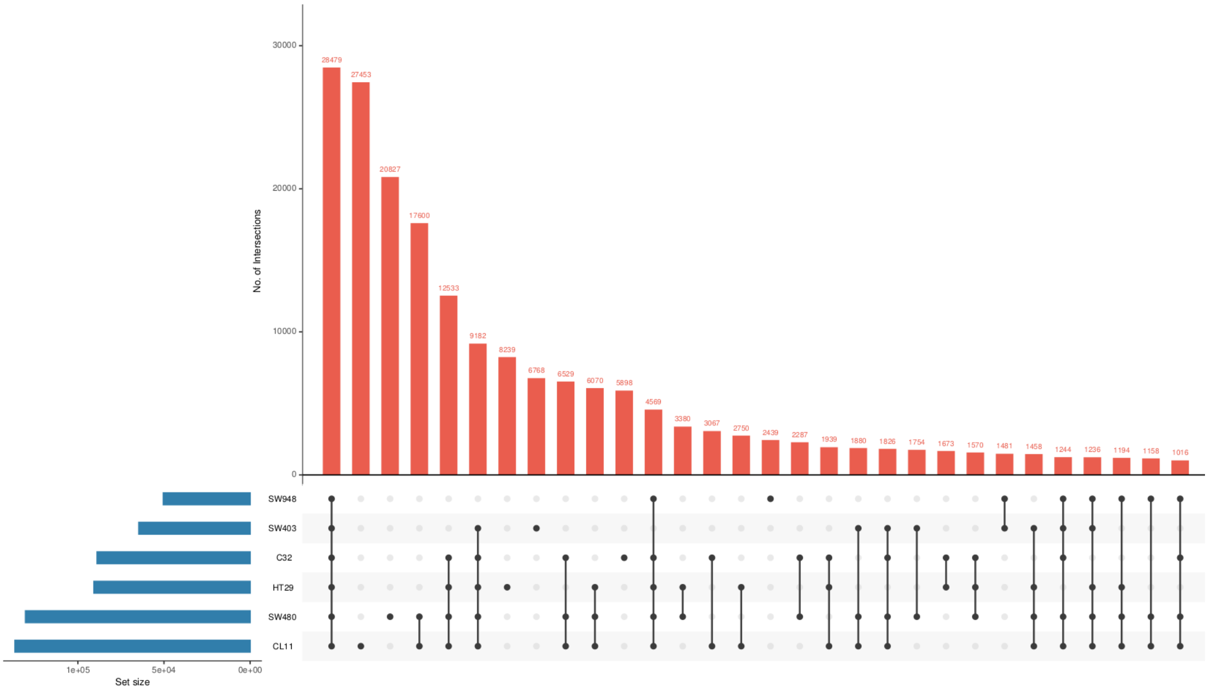

Supplementary Figure 9: UpSet plots of the peaks for the ATAC-seq, and ChIP-seq on CTCF, H3K4me1, H3K4me3, H3K27ac, H3K27me3, and H3K36me3 across the different cell lines. Plots generated using intervene[18].

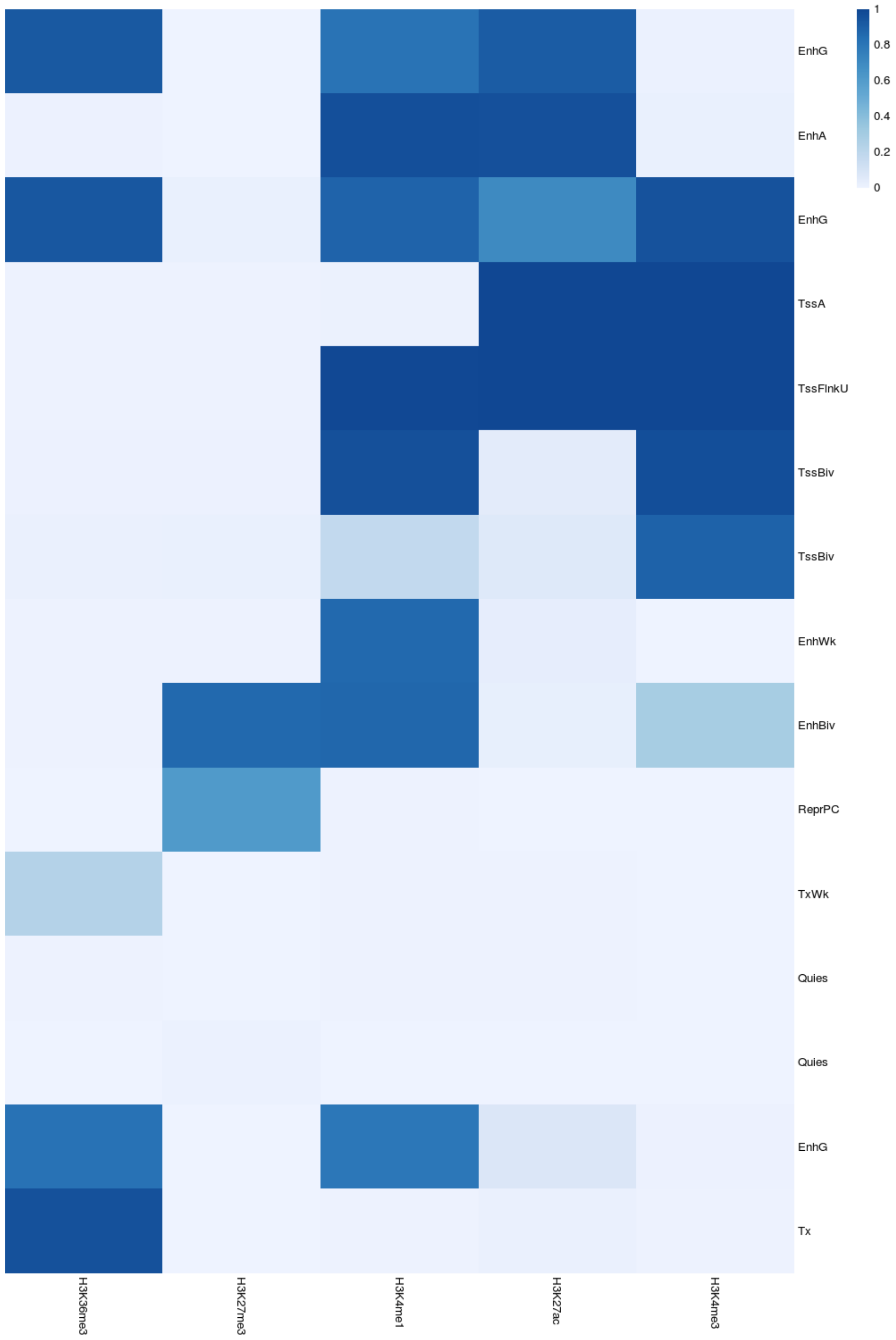

Supplementary Figure 10: Description of the identified states from the 15-state chromHMM model.

SUPPLEMENTARY REFERENCES

1. Schmidl, C., Rendeiro, A.F., Sheffield, N.C. & Bock, C. ChIPmentation: fast, robust, low-input ChIP-seq for histones and transcription factors. *Nat. Methods* **12**, 963-965 (2015).
2. Ewels, P. *et al.* The nf-core framework for community-curated bioinformatics pipelines. (Zenodo, 2022).
3. Corces, M.R. *et al.* An improved ATAC-seq protocol reduces background and enables interrogation of frozen tissues. *Nat Methods* **14**, 959-962 (2017).
4. Patel, H. *et al.* nf-core/atacseq: nf-core/atacseq v1.2.1 - Iron Centipede. (2020).
5. Krietenstein, N. *et al.* Ultrastructural Details of Mammalian Chromosome Architecture. *Mol. Cell* **78**, 554-565.e7 (2020).
6. Hsieh, T.-H.S. *et al.* Resolving the 3D Landscape of Transcription-Linked Mammalian Chromatin Folding. *Mol. Cell* **78**, 539-553.e8 (2020).
7. Durand, N.C. *et al.* Juicer Provides a One-Click System for Analyzing Loop-Resolution Hi-C Experiments. *Cell Syst* **3**, 95-8 (2016).
8. Nora, E.P. *et al.* Molecular basis of CTCF binding polarity in genome folding. *Nat. Commun.* **11**, 1-13 (2020).
9. Kaul, A., Bhattacharyya, S. & Ay, F. Identifying statistically significant chromatin contacts from Hi-C data with FitHiC2. *Nat. Protoc.* **15**, 991-1012 (2020).
10. Venev, S. *et al.* open2c/cooltools: v0.5.4. (Zenodo, 2023).
11. Lataretu, M. & Hölzer, M. RNAflow: An Effective and Simple RNA-Seq Differential Gene Expression Pipeline Using Nextflow. *Genes* **11**, 1487 (2020).
12. Zhang, M.J. *et al.* Polygenic enrichment distinguishes disease associations of individual cells in single-cell RNA-seq data. *Nat. Genet.* **54**, 1572-1580 (2022).
13. Tabula Sapiens, C. *et al.* The Tabula Sapiens: A multiple-organ, single-cell transcriptomic atlas of humans. *Science* **376**, eabl4896 (2022).
14. Elmentaite, R. *et al.* Cells of the human intestinal tract mapped across space and time. *Nature* **597**, 250-255 (2021).
15. Kundaje, A. *et al.* Integrative analysis of 111 reference human epigenomes. *Nature* **518**, 317-330 (2015).
16. Türei, D., Korcsmáros, T. & Saez-Rodriguez, J. OmniPath: guidelines and gateway for literature-curated signaling pathway resources. *Nat. Methods* **13**, 966-967 (2016).
17. Garcia-Alonso, L., Holland, C.H., Ibrahim, M.M., Turei, D. & Saez-Rodriguez, J. Benchmark and integration of resources for the estimation of human transcription factor activities. *Genome Res.* **29**, 1363-1375 (2019).
18. Khan, A. & Mathelier, A. Intervene: a tool for intersection and visualization of multiple gene or genomic region sets. *BMC Bioinformatics* **18**, 287 (2017).
