# Supplementary material for: Systematic prioritization of functional variants and effector genes underlying colorectal cancer risk
Source: Nat Genet. 2024 Sep 16;56(10):2104–11. doi: 10.1038/s41588-024-01900-w (PMC11525171; doi:10.1038/s41588-024-01900-w)
Supplement: Supplementary file 2 — Reporting Summary [file 41588_2024_1900_MOESM2_ESM.pdf]

nature portfolio

Corresponding author(s): Richard Houlston
Last updated by author(s): Jul 29, 2024

Reporting Summary

Nature Portfolio wishes to improve the reproducibility of the work that we publish. This form provides structure for consistency and transparency in reporting. For further information on Nature Portfolio policies, see our Editorial Policies and the Editorial Policy Checklist.

Statistics

For all statistical analyses, confirm that the following items are present in the figure legend, table legend, main text, or Methods section.

n/a	Confirmed	
☐	☒	The exact sample size (*n*) for each experimental group/condition, given as a discrete number and unit of measurement
☒	☐	A statement on whether measurements were taken from distinct samples or whether the same sample was measured repeatedly
☐	☒	The statistical test(s) used AND whether they are one- or two-sided<br>*Only common tests should be described solely by name; describe more complex techniques in the Methods section.*
☐	☒	A description of all covariates tested
☐	☒	A description of any assumptions or corrections, such as tests of normality and adjustment for multiple comparisons
☐	☒	A full description of the statistical parameters including central tendency (e.g. means) or other basic estimates (e.g. regression coefficient) AND variation (e.g. standard deviation) or associated estimates of uncertainty (e.g. confidence intervals)
☐	☒	For null hypothesis testing, the test statistic (e.g. *F*, *t*, *r*) with confidence intervals, effect sizes, degrees of freedom and *P* value noted<br>*Give P values as exact values whenever suitable.*
☒	☐	For Bayesian analysis, information on the choice of priors and Markov chain Monte Carlo settings
☒	☐	For hierarchical and complex designs, identification of the appropriate level for tests and full reporting of outcomes
☐	☒	Estimates of effect sizes (e.g. Cohen's *d*, Pearson's *r*), indicating how they were calculated

Our web collection on statistics for biologists contains articles on many of the points above.

Software and code

Policy information about availability of computer code

Data collection

No software was used for data collection

Data analysis

ABC v0.2.2: https://github.com/broadinstitute/ABC-Enhancer-Gene-Prediction
Akita v0.6: https://github.com/calico/basenji
ChromHMM v1.24: http://compbio.mit.edu/ChromHMM/
cooltools v0.5.4: https://github.com/open2c/cooltools
distiller-nf v0.3.4: https://github.com/open2c/distiller-nf
Enrichr 2023.06.08 release: https://maayanlab.cloud/Enrichr
FitHiC2 v2.0.8: https://github.com/ay-lab/fithic
GCTA-COJO v1.92.3: https://yanglab.westlake.edu.cn/software/gcta/#COJO
HapoReg v4.1: https://pubs.broadinstitute.org/mammals/haploreg/haploreg.php
intOGen 2020.02.01 release: https://www.intogen.org/
JuicerTools v1.22: https://github.com/aidenlab/JuicerTools
MAGMA v1.10: https://cncr.nl/research/magma/
META v1.7 : https://mathgen.stats.ox.ac.uk/genetics_software/meta/meta.html
Matrix eQTL v2.3: https://www.bios.unc.edu/research/genomic_software/Matrix_eQTL/
MELODI Presto (accessed 2024.04.03): https://melodi-presto.mrcieu.ac.uk/
MPRAflow v2.3.5: https://github.com/shendurelab/MPRAflow
MPRAnalyze v1.12.0: https://www.bioconductor.org/packages/release/bioc/html/MPRAnalyze.html
nfcore-atacseq v1.2.1 https://nf-co.re/atacseq/
nfcore-chipseq v1.2.1: https://nf-co.re/chipseq

OncoEnrichR v1.4.2.1: https://oncotools.elixir.no/
OncoScore v1.30.0: https://www.galseq.com/next-generation-sequencing/oncoscore-software/
PolyFun (downloaded 2023.09.13): https://github.com/omerwe/polyfun
RNAflow v1.4.1: https://github.com/hoelzer-lab/rnaflow
scDRS v1.0.1: https://github.com/martinjzhang/scDRS
susieR v0.11.92: https://github.com/stephenslab/susieR
SMR v1.3.1: https://yanglab.westlake.edu.cn/software/smr/#Overview
TOBIAS v0.14.0: https://github.com/loosolab/TOBIAS

For manuscripts utilizing custom algorithms or software that are central to the research but not yet described in published literature, software must be made available to editors and reviewers. We strongly encourage code deposition in a community repository (e.g. GitHub). See the Nature Portfolio guidelines for submitting code & software for further information.

Data

Policy information about availability of data

All manuscripts must include a data availability statement. This statement should provide the following information, where applicable:

- Accession codes, unique identifiers, or web links for publicly available datasets
- A description of any restrictions on data availability
- For clinical datasets or third party data, please ensure that the statement adheres to our policy

GWAS data are available from GWAS Catalog (accession no. GCST90129505). Cell line data have been deposited the European Genome-phenome Archive (EGA) under the following accessions: EGAD50000000294 (Micro-C), EGAD50000000295 (ChIP-seq, all marks), EGAD50000000296 (ATAC-seq), EGAD50000000297 (RNA-seq), EGAD50000000596 (MPRA). Annotation data for all the GWAS regions are available on UCSC Genome Browser (https://genome.ucsc.edu/s/philip.law%40icr.ac.uk/CRC%20GWAS%20annotation).
Single cell RNA-seq data were obtained from the Gut Cell Atlas (https://www.gutcellatlas.org/) and the Tabula Sapiens project (https://tabula-sapiens-portal.ds.czbiohub.org/). Transcription Factor binding was based on data from JASPAR (https://jaspar.genereg.net/). Functional annotations for the finemapping were obtained from the Alkes Price group (https://alkesgroup.broadinstitute.org/LDSCORE/). Histone marks in different tissues was obtained from the NIH Roadmap Epigenomics Project (https://egg2.wustl.edu/roadmap/web_portal/). eQTL data were obtained from PancanQTL (http://bioinfo.life.hust.edu.cn/PancanQTL/) and GTEx (https://gtexportal.org/). Literature mining was performed using data from the Semantic MEDLINE Database (SemMedDB, https://lhncbc.nlm.nih.gov/ii/tools/SemRep_SemMedDB_SKR.html), as implemented in MELODI Presto (https://melodi-presto.mrcieu.ac.uk/). Gene annotation data were obtained from the OmniPath (https://omnipathdb.org/), DoRothEA (https://saezlab.github.io/dorothea/), DepMap (https://depmap.org/), and Open Targets (https://www.opentargets.org/), as implemented in oncoEnrichR (https://oncotools.elixir.no/).

Research involving human participants, their data, or biological material

Policy information about studies with human participants or human data. See also policy information about sex, gender (identity/presentation), and sexual orientation and race, ethnicity and racism.

Reporting on sex and gender	N/A
Reporting on race, ethnicity, or other socially relevant groupings	N/A
Population characteristics	N/A
Recruitment	N/A
Ethics oversight	N/A

Note that full information on the approval of the study protocol must also be provided in the manuscript.

Field-specific reporting

Please select the one below that is the best fit for your research. If you are not sure, read the appropriate sections before making your selection.

☒ Life sciences ☐ Behavioural & social sciences ☐ Ecological, evolutionary & environmental sciences

For a reference copy of the document with all sections, see nature.com/documents/nr-reporting-summary-flat.pdf

Life sciences study design

All studies must disclose on these points even when the disclosure is negative.

Sample size	Observational study in cell lines, so sample size determination was not necessary
Data exclusions	Standard data QC was performed on all assays. Sequencing quality was assessed to ensure sufficient quality.<br>In the MPRA, we only retained reads that mapped perfectly to the barcode library, enforced using a CIGAR string of 230M. We additionally filtered the barcodes to those that contained all four allele specific probes (i.e. fwd_ref, fwd_alt, rev_ref, rev_alt), and additionally only

retained a barcode if there was a DNA read present with a corresponding RNA read in the same replicate.
For the ChIP-seq, ATAC-seq, RNA-seq, and Micro-C reads were removed if:
-reads mapped to blacklisted regions
-reads were marked as duplicates
-reads weren't marked as primary alignments
-reads were unmapped
-reads mapped to multiple locations
-reads contained > 4 mismatches
-reads had an insert size > 2kb
-reads mapped to different chromosomes
-reads arent in FR orientation
-reads where only one read of the pair fails the above criteria

Replication

For the ChIP-seq and ATAC-seq, each assay was performed with two replicates. For the RNA-seq, each cell line was performed with three replicates. For the Micro-C, each cell line was performed with eight sub-libraries. For the MPRA, each cell line was performed with three replicates. All replicates succeeded.

Randomization

Observational study, so randomisation was not necessary

Blinding

Observational study, so blinding was not necessary

Reporting for specific materials, systems and methods

We require information from authors about some types of materials, experimental systems and methods used in many studies. Here, indicate whether each material, system or method listed is relevant to your study. If you are not sure if a list item applies to your research, read the appropriate section before selecting a response.

Materials & experimental systems

n/a	Involved in the study
☐	☒ Antibodies
☐	☒ Eukaryotic cell lines
☒	☐ Palaeontology and archaeology
☒	☐ Animals and other organisms
☒	☐ Clinical data
☒	☐ Dual use research of concern
☒	☐ Plants

Methods

n/a	Involved in the study
☐	☒ ChIP-seq
☒	☐ Flow cytometry
☒	☐ MRI-based neuroimaging

Antibodies

Antibodies used

All antibodies obtained from Diagenode. 5ug of target antibody was added per 3-5 x 10^5 cell lysate.
Catalog Antibody lot
1.C15410196 H3K27ac Antibody - ChIP-seq Grade A1723-0041D
2.C15410195 H3K27me3 Antibody - ChIP-seq Grade A0824D
3.C15410194 H3K4me1 Antibody - ChIP-seq Grade A1862D
4.C15410192 H3K36me3 Antibody - ChIP-seq Grade A1845P
5.C15410003-50 H3K4me3 Antibody - ChIP-seq Grade A8034D
6. C15410210-50 CTCF Antibody - ChIP-seq Grade A2354-0010

Validation

All chip-seq grade antibodies were validated by Diagenode (Hologic, USA) and the details of validation experiments are provided in the links. For example H3k27ac (C15410196) validation was performed in HeLa cell lines by quantitative PCR using primer pairs for active promoters of EIF4A2 and ACTB as positive controls while TSH2B and MYT1 promoters were used as negative controls. Further details are available for each antibody using the relevant links.

https://www.diagenode.com/en/p/h3k27ac-polyclonal-antibody-premium-50-mg-18-ml; H3K27ac Antibody (Diagenode Cat# C15410196 Lot# A1723-0041D)
https://www.diagenode.com/en/p/h3k27me3-polyclonal-antibody-premium-50-mg-27-ml; H3K27me3 Antibody (Diagenode Cat# C15410195 Lot# A0824D)
https://www.diagenode.com/en/p/h3k4me1-polyclonal-antibody-premium-50-mg; H3K4me1 Antibody (Diagenode Cat# C15410194 Lot# A1862D)
https://www.diagenode.com/en/p/h3k36me3-polyclonal-antibody-premium-50-mg; H3K36me3 Antibody (Diagenode Cat# C15410192 Lot# A1845P)
https://www.diagenode.com/en/p/h3k4me3-polyclonal-antibody-premium-50-ug-50-ul; H3K4me3 Antibody (Diagenode Cat# C15410003-50 Lot# A8034D)
https://www.diagenode.com/en/p/ctcf-polyclonal-antibody-classic-50-mg; CTCF Antibody (Diagenode Cat# C15410210-50 Lot# A2354-00234P)

Eukaryotic cell lines

Policy information about cell lines and Sex and Gender in Research

Cell line source(s)	DSMZ: https://celldive.dsmz.de/<br>ECACC: https://www.culturecollections.org.uk/<br>EverCyte:https://evercyte.com/<br>ATCC: https://www.atcc.org/<br>SW403 (ACC294, DSMZ)<br>SW480 (ACC313, DSMZ)<br>SW948 (91030714, ECACC)<br>HT29 (ACC299, DSMZ)<br>CL11 (ACC467, DSMZ)<br>C32 (12022908, ECACC)<br>HCEC-1CT (CkHT039-0229, Evercyte)<br>HEK293T (CRL-11268, ATCC)
Authentication	All cell lines used are well characterised and established, and recently obtained from reputable vendors. We used whole genome sequencing using NGS to perform STR profiling to authenticate our cell lines.
Mycoplasma contamination	Routinely checked for Mycoplasma contamination kit (LOOKOUT MYCOPLASMA PCR DETECTION KIT , Sigma Aldrich , USA)
Commonly misidentified lines<br>(See ICLAC register)	No commonly misidentified cell lines were used in the study

Plants

Seed stocks	*Report on the source of all seed stocks or other plant material used. If applicable, state the seed stock centre and catalogue number. If plant specimens were collected from the field, describe the collection location, date and sampling procedures.*
Novel plant genotypes	*Describe the methods by which all novel plant genotypes were produced. This includes those generated by transgenic approaches, gene editing, chemical/radiation-based mutagenesis and hybridization. For transgenic lines, describe the transformation method, the number of independent lines analyzed and the generation upon which experiments were performed. For gene-edited lines, describe the editor used, the endogenous sequence targeted for editing, the targeting guide RNA sequence (if applicable) and how the editor was applied.*
Authentication	*Describe any authentication procedures for each seed stock used or novel genotype generated. Describe any experiments used to assess the effect of a mutation and, where applicable, how potential secondary effects (e.g. second site T-DNA insertions, mosiacism, off-target gene editing) were examined.*

ChIP-seq

Data deposition

☒ Confirm that both raw and final processed data have been deposited in a public database such as GEO.

☒ Confirm that you have deposited or provided access to graph files (e.g. BED files) for the called peaks.

Data access links<br>*May remain private before publication.*	https://ega-archive.org/datasets/EGAD50000000295
Files in database submission	All fastq and bed files for C32, CL11, HT29, SW403, SW480, SW948 on H3K4me1,H3K4me3, H3K27ac, H3K27me3, H3K36me3 and CTCF, as well as input. Each dataset has two replicates.
Genome browser session<br>(e.g. UCSC)	https://genome.ucsc.edu/s/philip.law%40icr.ac.uk/CRC%20GWAS%20annotation

Methodology

Replicates	For each cell line each anitbody capture Chipmentation experiment was performed in two replicates. This comprise six antibodies and an IgG and a Input control
Sequencing depth	Illumina Novaseq 6000, Single End sequencing, 100bp reads, Dual barcode (8bp,8bp), Sequencing depth varied from 30 million to 100 million reads.
Antibodies	All antibodies obtained from Diagenode<br># Catalog Antibody lot<br>1.C15410196 H3K27ac Antibody - ChIP-seq Grade A1723-0041D<br>2.C15410195 H3K27me3 Antibody - ChIP-seq Grade A0824D<br>3.C15410194 H3K4me1 Antibody - ChIP-seq Grade A1862D<br>4.C15410192 H3K36me3 Antibody - ChIP-seq Grade A1845P<br>5.C15410003-50 H3K4me3 Antibody - ChIP-seq Grade A8034D

	6. C15410210-50 CTCF Antibody - ChIP-seq Grade A2354-0010
Peak calling parameters	MACS broad peak
Data quality	As part of the nf-core chipseq pipeline, extensive QC is performed, including adapter trimming, filtering duplicate reads and poorly mapped reads
Software	nf-core chipseq pipeline summary:<br>Raw read QC (FastQC)<br>Adapter trimming (Trim Galore!)<br>Alignment (BWA)<br>Mark duplicates (picard)<br>Merge alignments from multiple libraries of the same sample (picard)<br>Re-mark duplicates (picard)<br>Filtering to remove:<br>-reads mapping to blacklisted regions (SAMtools, BEDTools)<br>-reads that are marked as duplicates (SAMtools)<br>-reads that arent marked as primary alignments (SAMtools)<br>-reads that are unmapped (SAMtools)<br>-reads that map to multiple locations (SAMtools)<br>-reads containing > 4 mismatches (BAMTools)<br>-reads that have an insert size > 2kb (BAMTools; paired-end only)<br>-reads that map to different chromosomes (Pysam; paired-end only)<br>-reads that arent in FR orientation (Pysam; paired-end only)<br>-reads where only one read of the pair fails the above criteria (Pysam; paired-end only)<br>Alignment-level QC and estimation of library complexity (picard, Preseq)<br>Create normalised bigWig files scaled to 1 million mapped reads (BEDTools, bedGraphToBigWig)<br>Generate gene-body meta-profile from bigWig files (deepTools)<br>Calculate genome-wide IP enrichment relative to control (deepTools)<br>Calculate strand cross-correlation peak and ChIP-seq quality measures including NSC and RSC (phantompeakqualtools)<br>Call broad/narrow peaks (MACS2)<br>Annotate peaks relative to gene features (HOMER)<br>Create consensus peakset across all samples and create tabular file to aid in the filtering of the data (BEDTools)<br>Count reads in consensus peaks (featureCounts)<br>Differential binding analysis, PCA and clustering (R, DESeq2)<br>Create IGV session file containing bigWig tracks, peaks and differential sites for data visualisation (IGV).<br>Present QC for raw read, alignment, peak-calling and differential binding results (MultiQC, R)
